# “Equity” in genomic health policies: a review of policies in the international arena

**DOI:** 10.3389/fpubh.2024.1464701

**Published:** 2024-12-20

**Authors:** Prabhathi Basnayake Ralalage, Tala Mitchell, Claire Zammit, Gareth Baynam, Emma Kowal, Libby Masey, Julie McGaughran, Tiffany Boughtwood, Misty Jenkins, Gregory Pratt, Angeline Ferdinand

**Affiliations:** ^1^Centre for Health Policy, Melbourne School of Population and Global Health, University of Melbourne, Carlton, VIC, Australia; ^2^Department of Community Services, Torrens University, Melbourne, VIC, Australia; ^3^Rare Care Centre, Perth Children’s Hospital, Perth, WA, Australia; ^4^Western Australian Register of Developmental Anomalies and Genetic Health WA, King Edward Memorial Hospital, Subiaco, WA, Australia; ^5^Alfred Deakin Institute, Deakin University, Melbourne, VIC, Australia; ^6^MJD Foundation, Alyangula, NT, Australia; ^7^Genetic Health Queensland, Royal Brisbane and Women’s Hospital, Brisbane, QLD, Australia; ^8^Australian Genomics Murdoch Children’s Research Institute, Parkville, VIC, Australia; ^9^Walter and Eliza Hall Institute of Medical Research, Parkville, VIC, Australia; ^10^Jawun Research Centre, CQUniversity, Brisbane, QLD, Australia

**Keywords:** policy, genomic, genetic services, equity, core concept, vulnerable groups

## Abstract

**Introduction:**

The field of genomics is rapidly evolving and has made significant impact on the diagnosis and understanding of rare and genetic diseases, in guiding precision medicine in cancer treatment, and in providing personalized risk assessment for disease development and treatment responses. However, according to the literature, there is widespread socio economic and racial inequities in the diagnosis, treatment, and in the use of genomic medicine services. This policy review sets out to explore the concept of equity in access to genomic care, the level of inclusion of equity and how it is addressed and what mechanisms are in place to achieve equity in genomic care in the international health policy.

**Methods:**

A systematic search for genomic policies was conducted using 3 databases. In addition, General and Specific Policy Repositories, Global Consortia in Genomic Medicine, WHO Collaborating Centers in Genomics, Australian Genomics, Public Policy Projects, Global Genomic Medicine Consortium (G2MC), G2MC conference Oct 2023 and National Human Genome Research Institute databases were searched using the inclusion and exclusion criteria. Seventeen policies were selected and analyzed using the EquiFrame.

**Results:**

The Core Concept of access is highly cited in most of the selected policies. The CCs that are covered to a lesser degree are participation, quality, coordination of services, cultural responsiveness and non-discrimination. The CCs of liberty and entitlement are not addressed in any of the selected policies. The coverage of vulnerable communities in the policies varies from country to country.

**Discussion:**

Genomic health science is rapidly evolving and presents a major challenge for policies to remain current and effectively address new discoveries in the field. There is a relative dearth of policies that focus on clinical genetic services which may reflect a gap in policy and policy research translation and implementation. Recommendations for countries, irrespective of their economic and social contexts, include conducting regular policy reviews to accommodate the advances in genomics field and inclusion of specific mechanisms to achieve equity in genomic health. Insights and experiences in achieving healthcare equity in HICs and LMICs can offer valuable lessons for each other.

## 1 Introduction

The field of genomics has rapidly evolved, significantly influencing clinical medicine (1). This evolution has led to advancements such as the diagnosis and understanding of rare and inherited diseases; guiding precision medicine in cancer treatment, and providing personalized risk assessment for disease development and treatment responses. However, despite widespread availability of genomic research findings, persistent socioeconomic and racial disparities exist in the diagnosis, treatment and usage of genomic medicine services (2). Inequitable access to genomic medicine and information, coupled with healthcare systems’ unpreparedness to offer genomic services universally, contribute to these disparities (1, 3).

Equity in genomic medicine is defined as the fair and equal application of genomic knowledge, ensuring everyone has access to services like testing and counselling, and that the implementation of genomic medicine is impartial (4). Researchers have argued that to address future equity in genomics we must first look at populations that have until now been left behind by the benefits of genomic medicine (4). There is substantial evidence to demonstrate that racial and ethnic minorities, Indigenous communities and rural residents have notably less access to genetics health services (5, 6). For instance, a report by the United Nations highlighted the severe inequity faced by the Australian Aboriginal population in accessing health care (7). In Australia, reporting of Indigeneity is delayed, inconsistent and fragmented (8) which affects Indigenous Australians’ access to the health benefits and their representation in health data (39). Despite recognition of equitable healthcare as a fundamental human right, various social and health system barriers impede equitable access to genomic-integrated healthcare (9, 41). Globally, diverse, marginalized and minority populations are under-represented at genomic health services, even though they have a higher prevalence of certain conditions and a strong demand for inclusion in clinical benefits (10). Hence, offering inclusive, accessible and universal services is crucial to extending these clinical benefits across all populations (11, 12).

The field of genomic medicine is grappling with significant challenges in meeting its responsibilities to ensure equitable access to genomics. For instance, research examining equity in genomic health service use among Indigenous Australians suggests that there is a three-fold under-representation of Indigenous Australians, despite demand (13). This disparity is further evidenced by the Better Indigenous Genetic (BIG) Health Services study, which highlights profound inequities in both the provision and patient experience of clinical genetic services (6).

The issue of inequitable access is not isolated but a global concern. Research from various regions shows that these disparities are widespread, and addressing them requires a fundamental redesign of healthcare systems. Increasing genomic awareness among people in lower socio-economic groups and culturally diverse and Indigenous populations is crucial. Strategies to enhance access to genomic services must be tailored to fit different contexts but are universally needed (14, 21).

This is the first evaluation of genomic policy that identifies the level of inclusion of the concept of equity and its shortfalls. This policy review focuses on the concept of equity in access to genomic care, the level of inclusion of equity and how it is addressed and what mechanisms are in place to achieve equity in genomic care in the international health policy.

## 2 Health equity and right for health

“The essence of global health equity is the idea that something so precious as health might be viewed as a right.”

-Paul Farmer

Paul Farmer’s words denote the intrinsic connection between the idea of the right to health and health equity. Human rights and health equity are profoundly linked to each other (15). His words also indirectly encapsulate the notion of health equity as a challenging exercise and the inadequacy of the current global health systems to achieve it. While demonstrating that both human rights and equity strive for the same aim--equal opportunity--Braveman and Gruskin further argue that the concept of human rights gives a universally applicable framework for the attainment of health equity (2003). Recent genomic discoveries and research have contributed to improved health outcomes and reduced morbidity and mortality. However, benefits of genomic discoveries are not distributed equally to all groups in a population. There are multiple socio-economic, geographical and cultural barriers that exist for equal provision and access to genomic health care in different contexts and this policy analysis explores how equity is embedded in global genomic health policy. In general, achieving equity has proved to be an evasive objective in public policy not only in health but also in other areas such as education (16) and public administration (17).

Equity is a key principle of the concept of “health for all” by United Nations that was brought forward by the Alma Ata Declaration (47) four decades ago. WHO defines health equity as ‘the absence of unfair, avoidable, remediable differences among groups of people’ (40) even though differences exist between the groups in the form of gender, sexual orientation, ethnicity, wealth, residence/ geographic location. Equity has been defined as having equal access to available care for equal need, equal utilization of services for equal need and equal quality of care for all [(18), p. 434]. In an attempt to define the meaning of equity in the health arena, in the 90s Whitehead affirms that equity aligns with seven principles: (i) Equity policies must focus on improving living and working conditions; (ii) Equity policies must be directed towards enabling healthier lifestyle; (iii) Equity policy must focus on engaging community participation and decentralization of decision making power; (iv) Equity policies must assess the impact on health by policies in all other sectors, especially the impact on health of Vulnerable Groups; (v) Equity policies must have mutual concern and control at the international level; (vi) Equity policies must assure universal access to high quality health care; and (vii) Equity policies should be based on relevant research, monitoring and evaluation (18).

Two decades after the publication of these health equity principles, policy researchers show the importance of narrowing down these broad principles and affirm the importance of making a distinct and explicit commitment to achieve equity in health with specific objectives. Mannan et al. (19) argue that governments’ action must be pro-equity. Drawing from EQUINET, they affirm that equity in health policy can be referred to as a “propitious political message” (p. 7) that is intended to bring social cohesion and solidarity through concrete political steps to provide mechanisms to protect the health of the poor. Equity is a cornerstone of policy making and its achievement depends on what the decision makers consider as priority health issues and the selection and inclusion of populations that most deserve attention (19). We delve into the details of how political leadership becomes a main driving force of achieving health equity in our next publication that focuses on the experiences and perceptions of achieving equity in genomic health of the policy makers in the international contexts.

### 2.1 EquiFrame to assess equity in policy

In the development of EquiFrame, Mannan et al. (19) drew heavily from Braveman and Guskin’s advocacy for the routine and systematic application of equity and human rights approach to health sector actions (2003). They also state that the human rights approach facilitates the analysis of policy from a range of diverse perspectives. The EquiFrame was developed using perspectives from human rights, the right to health and vulnerability. This framework facilitates the exploration of the inclusion of equity in genomic policy and it gives us a clear view of the level of commitment these policies employ to achieve health equity. Mannan et al. make reference to 21 Core Concepts (CCs) that are focused on the individual and the collective in relation to “principles of universal, equitable and accessible health services” [(19), p.13].

### 2.2 Contextualizing genomic health policy

In general, policy is influenced by factors such as understanding and framing of health; use of evidence; contextual priorities and political ideologies; systems; and leadership (20). Inequity in access to genetic health care among diverse populations has been attributed to inadequate integration of genomics policies into the general health systems and policy gaps in genomic health itself (21, 46). These policy challenges need to be addressed through better understanding of the barriers for integration of genomics into policies, through policy frameworks that are informed by population health priorities, and through engagement with underserved communities, health care providers and policy makers (21, 22, 45, 46). Even in many parts of the developed world, it appears that genomic policy has not caught up with the developments of genomic research and its benefits.

Khoury et al. (5) state that an agenda of health equity in genomics needs to go beyond clinical research and should include health policy. The authors argue that what they term as a public health agenda is needed to address the disparities in implementation of genomics in different populations. To address these disparities, they state that this public health action needs to focus on, (1) population-specific needs and outcomes assessment, (2) policy and evidence development and (3) assurance of delivery of ethical and effective interventions. They further argue that absence of concerted public health action and further advancements of genomics will only widen the equity gap. The current review that explores the inclusion of equity concepts in existing genomic health policy and identify barriers and challenges to equity-informed genomic policy, aims to support the type of public health agenda that Khoury et al. (5) advocate for.

### 2.3 Concept of equity

Equity in genomics has been defined as the successful implementation of genomic medicine where all populations have equitable access to effective affordable genomic medicine which includes diagnosis, treatment and prevention strategies (4). Genomic health policy has a crucial role to play in ensuring the equitable distribution of and access to genomic medicine. As there are currently many efforts being made to translate genomic medicine benefits into health care services, it is an important time for policymakers along with researchers, funders and administrators to capitalize on this progress to ensure equitable distribution of health benefits (4).

In such an exercise, first it is important to understand whether and how equity is integrated into genomic policy and what strategies policymakers have already implemented to ensure equity. In this policy analysis we will use the EquiFrame framework designed by Mannan et al. (19) to rate and analyze the level of incorporation of the concepts of inclusion and equity in genetic health policies internationally.

## 3 Objectives

The aim of this policy review is to identify approaches that will support the development of policy at a health system level to enable the equitable distribution of benefits of genomic health services to marginalized populations including First Nations, as the field continues to develop. To do this, the policy analysis explored the level of alignment with concepts of equity in global genomic policies in relation to making genomic testing and medical benefits accessible to everyone and identified exemplars of equitable policy approaches to genomics services. Key factors that contribute to equitable genomic services in global genomic policies are identified and recommendations provided for equitable genomic health policy development; both in relation to the *content* of equitable genomic health policy and with regards to equitable *processes* in policy development.

In this policy analysis we will explore the following research questions.

1. To what extent are the key concepts of *equity* (as defined by EquiFrame) incorporated in genomic health policies, particularly in relation to Indigenous and other vulnerable populations?
2. Where are the gaps or shortfalls in equitable policy development?
3. What mechanisms are outlined in genomic health policies to monitor outcomes against equity principles?

## 4 Methods

The first step of conducting the policy analysis was the search for active genomic health policies internationally. The below table shows the inclusion and exclusion criteria (Table 1).

**Table 1:** Inclusion and exclusion criteria.

Inclusion criteria	Policies published in English and French Policies that are currently active or the last policy to be active Relevant to genomic medicine and health delivery at a national/population level
Exclusion criteria	Laws, legislations and acts Policies about genomic data Integration and implementation roadmaps Demonstration projects and initiatives Population studies

Three databases were used (Medline Ovid, Scopus and CABI Global Health) for the search and in addition General and Specific Policy Repositories (Genomic Medicine Policy), Global Consortia in Genomic Medicine, WHO Collaborating Centres in Genomics, Australian Genomics, Public Policy Projects, Global Genomic Medicine Consortium (G2MC), G2MC conference Oct 2023 and National Human Genome Research Institute (Database on genetic policy and laws) databases were searched.

**Figure 1:**
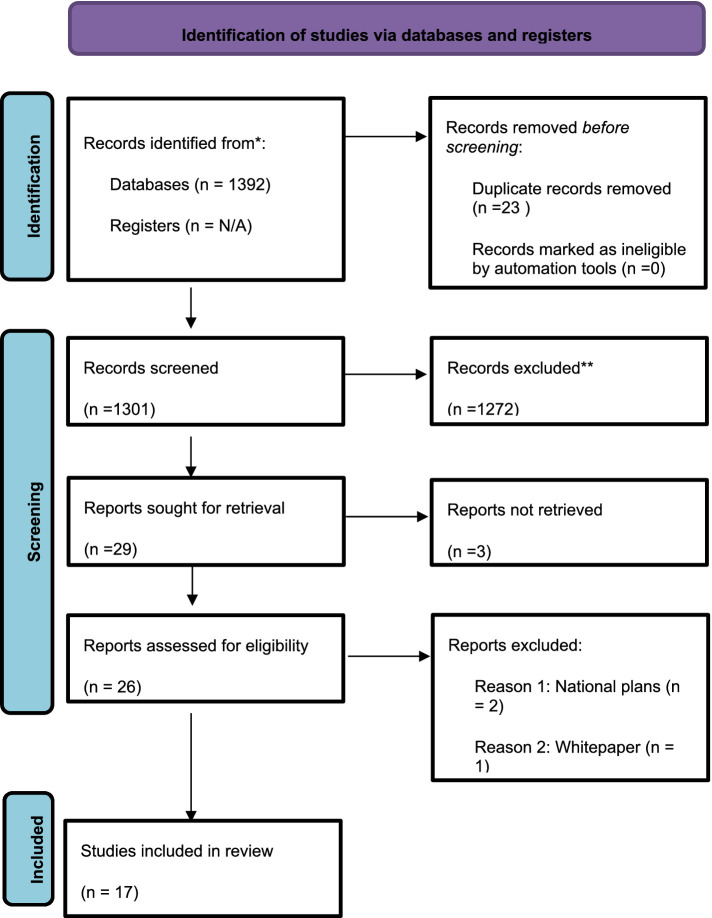
The PRISMA diagram.

The Preferred Reporting Items for Systematic Reviews and Meta-Analyses (PRISMA) diagram below demonstrates the process we followed in searching and selecting the policies^1^ (Figure 1).

We reviewed 17 genomic policies in Europe, Southeast Asia, Canada and Australia. Policy is defined by Howlett and Cashore (23) as the “actions that contains goals and the means to achieve them, however well or poorly identified, justified, articulated and formulated” (p. 17) while Dye (42) sees it simply as the actions that a government decides to take and not take. We have employed EquiFrame to analyze the extent to which policies in genomics have employed the concepts of equity, social inclusion and human rights (19). EquiFrame has been developed by Mannan et al. (19) as a framework that can be used to assess the degree to which equity has been addressed in policies and policy related documents. EquiFrame views social inclusion and human rights as key components of equity. Furthermore, EquiFrame enables the evaluation of the degree to which a health policy demonstrates commitment to 21 CCs of human rights and to 12 Vulnerable Groups (VGs), guided by the ethos of universal, equitable and accessible health services. According to the EquiFrame authors, this framework can be customised to the requirements of the purpose of the analysis (19).

Accordingly, the EquiFrame framework (a) defines Core Concepts, (b) identifies the key questions and key language on which the concept is based, (c) identifies Vulnerable Groups, and (d) provides a data extraction matrix to chart the analyzed documents. The EquiFrame Matrix lists the 21 Core Concepts along the vertical axis, and 12 Vulnerable Groups along the horizontal axis.

For instance, VGs and CCs may be added or removed to suit specific requirements, political, cultural or other contextual interests or constraints. In this analysis, Indigenous groups are added to the VGs which makes the total of 13 Vulnerable Groups that each selected policy is assessed.

Accordingly, the matrix was developed with the 21 CCs along the vertical axis and 13 VGs along the horizontal axis for each of the selected policy document.

### 4.1 Scoring

Each policy was assessed against the 21 Core Concepts of the EquiFrame framework and received a score from 1 to 4 for each Core Concept. This is a rating of the quality of commitment to the Core Concept within the policy document:

1 = Core Concept mentioned.

2 = Core Concept mentioned and explained.

3 = Specific policy actions identified to address the Core Concept.

4 = Intention to monitor concept was expressed.

If a Core Concept was not relevant to the document context, it is stated as not applicable.

In each document, the presence of Core Concepts is addressed for each Vulnerable Group that is identified in the policy. If no Vulnerable Group was mentioned, but a Core Concept addressed the total population (e.g., “all people”), the Core Concept was scored as *Universal.* The total number and scores mentioned in the Core Concepts and Vulnerable Groups is calculated for each document, across all countries the policies originate from.

Inter-rater reliability was achieved through comparing separate evaluations between two raters. Two raters independently reviewed the EquiFrame analysis. Regarding inter-rater reliability for the application of EquiFrame to identified policies, percentage agreement was calculated regarding all summary indices for each policy.

### 4.2 Summary indices for EquiFrame

Four Summary Indices of EquiFrame are outlined below. Each policy is rated according to the percentage they received for addressing and the quality of the Core Concepts and Vulnerable Groups.

1. Core Concept Coverage: The policy is examined with respect to the number of Core Concepts mentioned out of 21 Core Concepts identified; and this ratio was expressed as a rounded-up percentage. In addition, the actual terminologies used to explain the Core Concepts, within each document, is extracted to allow for future qualitative analysis using NVivo and cross-checking between raters.
2. Vulnerable Group Coverage: The policy was examined with respect to the number of Vulnerable Groups mentioned out of 13 Vulnerable Groups identified: and this ratio is expressed as a rounded-up percentage. In addition, the actual terminologies used to describe the Vulnerable Groups were extracted.
3. Core Concept Quality: The policy is examined with the respect to the number of Core Concepts within it that are rated as 3 or 4; that is, as either stating a specific policy action or intention to monitor action. This is also transferred to a percentage rate. When several references to a Core Concept were found to be present, the top-quality or highest score received is recorded as the final quality scoring for the respective Core Concept.
4. Overall Summary Ranking: Each document is given an overall summary ranking in terms of it being Low, Moderate or High standing according to the following criteria:
  - High = if the policy achieved ≥50% on all the three scores above
  - Moderate = if the policy achieved ≥50% on two of the three scores above
  - Low = if the policy achieved <50% on two or three of the three scores above

## 5 Results

A total of 17 policies were selected from the desktop search of literature. The policies came from Australia, Hong Kong, Thailand, India, United Kingdom, Denmark, Finland, Italy and Canada. One of the 17 policies is an international genomic policy developed by the International Rare Disease Consortium.

Out of the 17 policies, most rated reasonably high (around or above 50%) for the Core Concept coverage. A smaller number of policies scored reasonably for the Vulnerable Group coverage. Indigenous populations, people from culturally and linguistically diverse backgrounds and people living remotely were the three main Vulnerable Groups addressed. Few policies scored reasonably for the Core Concept Quality, which was calculated from the categories of either stating a specific policy action, or intention to monitor action in the policy.

### 5.1 Inclusion of core concepts

In this section only the concepts that are most frequently addressed across the 17 policies are reported and summarized. In each section, a comment is made on the Core Concept’s EquiFrame analysis rating, with a summary of how the Core Concepts are included in the policies and an attempt to compare policies.

#### 5.1.1 Access

The Core Concept of ‘access’ is the most widely addressed concept related to equity in the selected policies. Access was addressed in 14 out of 17 policies (82%). The ratings that are given in the EquiFrame for the Core Concept of access in the 14 policies varies between 1 and 2 (between ‘access’ being stated in the policy to ‘access’ being explained).

Out of these 14 policies, the genomic policies of Western Australia (2022), New South Wales Genomics Strategy (2020) and in the United Kingdom, one of rare diseases (2021) and the National Health Services genomic policy (2022) mention the necessity of equitable access to genomic medicine in varying degrees.

##### 5.1.1.1 Multi dimensionality and priority areas of access

Multi dimensionality of access is an aspect that is acknowledged in most of these policies. For example, the Australia National Health Genomic Policy Framework 2018–2021 details the multidimensionality of access as it includes factors such as location, cost, availability and cultural acceptability which would drive the ability to access genomic care especially of vulnerable populations. For example, the policy identifies the dimension of culturally secure, appropriate and responsive genomic services as a priority in relation to addressing the problems of access to genomic services of the Aboriginal and Torres Strait Islander populations.

Under Western Australia Genomic Health Policy (2022), ‘access’ is also referred to as the health consumers’ ability to access educational material and increased awareness of the applications of genomic innovations in order to receive benefits from genomic healthcare.

In some policies there are specific priority areas that are identified related to access. Both in the WA policy and the UK Rare diseases Framework, the use of novel and advanced technology and digital tools is referred to as a priority to increase remote access for patients in WA and the UK. According to the two policies, improving access to specialist care and treatment entails innovation and commitment for innovation and collaboration of the health system with stakeholders.

Providing access to vulnerable populations is also identified as a strategic priority in some policies. WA policy recognizes that Indigenous populations, people from culturally and linguistically diverse backgrounds and communities living in rural and remote regions need to be given priority when championing access to value based genomic services. In addition, the Australia National Health Genomics Policy Framework 2018–2021 too identifies the principle of equity in access as an area of strategic priority specifically related to vulnerable populations (p. 4). The UK Rare Diseases Framework includes the Core Concept of access mainly in relation to Black, ethnic minority communities and patients living in remote parts of the country.

##### 5.1.1.2 Increasing access through the mainstream health care

Both the policies of WA and NHS in the UK recommend the integration of genomic services to the mainstream health system to reinforce equitable access of genomic services.

For example, the vision of the UK genomic policy Accelerating Genomic Medicine in the NHS (43), is that treatment is accessible to all, as part of routine care at NHS. This policy sets out to achieve the vision through four priority areas; (1) by embedding genomic testing and medicine across NHS (2) by providing equitable genetic services for improved outcomes (3) by ensuring genomic data could be interpreted and used by other diagnostic data (4) by evolving the service through cutting edge science (so that the patient can make use of rapidly evolving improvements in the science).

The WA policy also identifies that integration of genomic care in the mainstream health care is an important strategy to increase access for the growing demand for genomic health care. Ensuring that WA health systems have processes, expertise and infrastructure in place to evaluate and implement continuous advances in genomic health services in the mainstream health care are identified as important strategies to increase access to genomic health services.

##### 5.1.1.3 Challenges to access

Some policies acknowledge challenges specifically related to access to genomic health care. For example, the WA policy recognizes continuing mistrust within Indigenous communities towards the healthcare system and genetic testing due to a historical lack of transparency and culturally appropriate ways of obtaining consent and culturally inappropriate uses of genomic data as a barrier to access.

The UK Rare Diseases Framework (2019) identifies the availability of limited data as a challenge to providing equitable access to genomic health services to patients with rare diseases. Another challenge according to the policy NHS policy (2022) is to find the balance between the provision of treatment for all patients with the need and the limited and fixed resources (p. 15).

As a solution to address some of these challenges, WA policy points to the importance of the health system being cognizant of the need for cross sector training, educational requirements and working arrangements across private, public and not for profit genomic service sectors to reinforce equitable access.

#### 5.1.2 Participation

The Core Concept ‘participation’ was addressed in genomic policies of Australia, UK, Canada, Hong Kong and India to varying degrees. Twelve out of the 17 genomic policies (70.5%) included participation as a Core Concept. Participation in them is addressed in two distinct areas: participation of the community in genomic research and community participation in genomic policy development and implementation. Participation of the community in genomic research is not in the scope of this review. In the latter category, the key question posed regarding the Core Concept of participation in the EquiFrame is if the policy supports the right of vulnerable groups to participate in the decisions that affect their lives and enhances their empowerment. The ratings of the 12 policies vary between 1 and 3 (from naming the Core Concept to detailing methods to achieve the Core Concept).

##### 5.1.2.1 Participation of vulnerable groups

UK policies of Rare Diseases Framework and the ‘Accelerating Genomic Medicine in the NHS’ (43) seem to take lead in acknowledging the importance of participation of vulnerable populations in the development of genomic policy.

A UK genomic policy that gives a high degree of attention to patient and community participation and collaboration is the Rare Diseases Framework. Although participation is not mentioned explicitly, patient and community voices and lived experience are an underpinning theme to achieve the framework’s four key priorities. It acknowledges that patients’ voice is at the center of decision-making in relation to all aspects in genetic care and service delivery, including policymaking. It focuses on the inclusion of patient representatives from Black, ethnic minority and disadvantaged communities (p. 17).

The UK policy ‘Accelerating Genomic Medicine in the NHS’ (43) embeds patient and public participation as a core principle and a key determinant in the design and of the success of the NHS Genomic Medicine Service (GMS) (p.15). The policy describes a strategy of achieving participation through multiple avenues and includes separate sets of recommendations to health providers, healthcare professionals, patients and patient groups to increase participation in genomic healthcare and research at all levels of NHS GMS. The role of health providers under the policy includes identifying unmet needs and inequalities and providing access for patients under their care to participate in genomic clinical trials and projects as well as genetic testing offered by NHS. The policy also supports increased patient and patient groups’ involvement in NHS GMS national and regional governance structures at all levels and promotes the role of the NHS in supporting such involvement. Thus, participation of patients and patient groups is included in this policy as an action to be taken in the development of governance policies.

Further, even though the UK genomic policy, Genome UK: the Future of Healthcare, refers to the Core Concept of participation only in relation to genomic research, it acknowledges an important barrier for the diversity of genomic datasets that exists in racially different communities. Participation in the policy is emphasized as an important step to achieve genomic equity of access and diversity (p. 39). The policy recognizes that the distrust and suspicion of Black Caribbean and ethnic minority communities towards genomic research and the assumptions of health professionals about ethnic minority population are possible barriers for participation.

The Canadian genomic policy too, focuses on participation of vulnerable populations in developing genomic policy only to a limited extent. Strategic Plan 2022–2027: Sequencing our future: A Vision for a Healthier Future (44) outlines four commitments: (1) enabling genomic medicine; (2) improving genetic disease diagnosis and therapies; (3) embracing diversity, inclusion and Indigenous rights; and (4) strengthening the community (capacity building). Participation and co-design are only mentioned in relation to genetic research under commitment 1 (p.11). Participation in clinical genetic services is not the focus here.

##### 5.1.2.2 Participation of consumers in policy implementation

Participation in the Australian genomic policies are not specifically mentioned in relation to the engagement of any specific vulnerable population.

Within Australian genomic policies, in the NSW Health Genomic Strategy: Implementation Plan 2021–2025, participation of consumers is incorporated in the implementation activities of the policy. Rather than consumers participating to co-develop the policy, it consolidates key actions that need to be taken to achieve the genomic strategy through a participatory and co-design approach with the key stakeholders. The key actions proposed in the strategy which incorporates the participation of the consumers are embedding, scaling and sustaining multidisciplinary clinical genomic models of care; developing and testing tools to support triage and referral pathways and to develop tools to translate genomic research into clinical practice appropriately and consistently; continuing to monitor requirements for data management, governance and access levels; developing guide to standards of genomic products that incorporates consumer needs and experiences; developing an electronic genomic tracking system; implement and integrate test result reporting with existing systems; establishing digital consent requirements for genomic testing; build and test digital consent for clinical genomic use; integrating patient and health professional educational resources with digital consent; developing educational website design to meet consumer requirements; redesign the educational resources portal; developing training needs assessment tools for workforce; and using workforce and educator champions support to deliver clinical genomics use (2021, p.5–7).

Within the Australia National Health Genomics Policy Framework 2018–2021, the terms ‘engagement’ and ‘participation’ are used interchangeably. The Framework recognizes the importance of consumer participation in effective health care delivery and conceptualizes individuals and families as active and engaged partners with the genomics health care delivery team. The policy framework also identifies stakeholder engagement as a key enabler of implementation success. The ‘Genetic and Genomic Healthcare for Victoria 2021: Improving the Health and Wellbeing of Victorians’ policy also integrates the Core Concept of participation in a universal sense.

‘Bringing innovation to life: Strategic Vision’ (2019) outlines the mission and vision of Genome Canada. Even though this is primarily relevant to researchers and research processes under Genome Canada, the policy also discusses service delivery to a limited extent. The Core Concept of participation and engagement is described as concepts through:

1. supporting research on implications of genomics in society.
2. working with stakeholders to develop and implement genomic strategies.
3. communicating trusted information of genetics to stakeholders.
4. contributing to a national dialogue on the intersection of genomics and policy (p. 23).

#### 5.1.3 Integration

In the EquiFrame manual the Core Concept of integration means integrating genomic care for Vulnerable Groups in the main or general healthcare system rather than having specialized services dedicated for Vulnerable Groups. Mannan et al. (19) argue that when incorporating the concept of integration meaningfully in policy, one must first investigate if the policy bars the vulnerable communities in participating in mainstream healthcare. The authors further argue that dedicated services for vulnerable populations could have inadvertent effects such as stigmatization, be at the risk of losing funds, become less desirable for health professionals and therefore face the risk of losing quality of care (p.54). This analysis demonstrates that the CC of integration in relation to including vulnerable population.

Overall, out of the total number of 17, 12 policies in the review either did not focus on integration or used the CC of integration in a manner that did not align with the meaning of the CC as outlined by Mannan et al. (19). The term ‘integration’ in these policies is used to denote the integration of genomic data and research in the genomic services; this is not within the scope of this review.

Five policies from Australia, UK and Denmark address the CC of integration of genomic services in the mainstream general healthcare services in varying degrees and detail. What these five policies demonstrate in common is the importance of integrating genomic clinical services in the general healthcare services in the delivery of person centered, equitable genomic care. Only two policies specifically address integration in the healthcare system with the intention of addressing the needs of vulnerable populations. While the policies from the UK emphasize the strong platform that NHS provides the Genomic Medicine Service (GMS) in the delivery of equitable, effective and sustainable genomic healthcare for the UK population, Australian genomic policy clearly demonstrate its commitment to integrate genomic services into general healthcare services.

Accelerating Genomic Medicine in the NHS recognizes that NHS is in a strategic position to implement genomic healthcare as a nationally organized locally delivered service. The policy points out that Genomic Medicine Service (GMS) of NHS is an integrated service model which have brought together genomic testing, clinical genetics and other services, research and innovation to deliver the benefits of genomics to all NHS patients (p. 16). The NHS policy states that the development of an integrated service model of genomic service via primary and community care is specifically focused at providing services for unmet needs and undiagnosed populations.

The UK Rare Diseases Framework too echoes this statement of how Genomic Medicine Service is integrated into the frontline healthcare service of NHS.

Australia’s National Health Genomics Policy Framework (2018–2021) focuses on the CC of integration in relation to integrating genomic healthcare in the national health system. The policy also acknowledges that the integration of the genomic services in the national health system is dependent on the acceptance of this by the community and the level of confidence that the community has on the genomics (p. 1). Even though it does not mention vulnerable populations in relation to integration, the policy views integration of genomic services in the national health system as a mechanism to address inequity and also recognizes the risk it still presents for potential discrimination of people.

#### 5.1.4 Quality

The Core Concept of quality has been recorded in 10 policies (58%) from Australia, Canada, Thailand, Hong Kong, Italy and Denmark. In most of these policies, ‘quality’ was the highest rated Core Concept that was discussed about in a universal sense.

##### 5.1.4.1 The features and definitions of quality

Adherence to quality and safety when promoting public trust in genomics in health care is considered to be the main underlying principle in the WA genomic strategy. Genomic policies around the world characterize quality in different ways. Quality referred to in genetic tests and services are both included in this review as they are both intrinsically related.

One common trend we see across these policies is that achieving high quality in genomic care is seen to be obtainable through a person-centered approach and highly skilled healthcare workforce.

For example, the WA genomics strategy characterizes health care quality as the result of a person and family centered approach along with better health outcomes, improved safety, cost effectiveness and consumer satisfaction (p.18).

High quality genetic healthcare service in the Genetic and Genomic Healthcare in Victoria (2021) is characterized by a skilled healthcare workforce that is able to build trust with the public and raise awareness of benefits and limitations of genomic care and on the growth and development of knowledge in the area (p. 12).

Proven high quality clinical services, safety, clinical utility and effective safe use of genomic data are related to quality in the genomic policy ‘Strategic Development of Genomic Medicine in Hong Kong (p. 39). Quality and efficiency are addressed specifically in relation to genetic tests and genetic services.

##### 5.1.4.2 The ways to achieve quality

The selected policies name strategies such as strengthening the overall health system, implementing high-quality genetic testing and implementing quality assurance mechanisms in all components of genomic services to improve quality in specific countries. For example, the Genetic and Genomic Healthcare in Victoria (2021) views strengthening the healthcare system as a key component of quality service provision.

In the Danish genomic policy 2020–2021, the country’s ability to provide high-quality genetic analysis to all patients irrespective of where they live is the outcome of continuous work on personalized medicine in Denmark, its consolidation and streamlining of clinical activities and supportive infrastructure (p. 8).

In the policy Genome British Columbia (2015), quality is mentioned as a requirement for successful clinical implementation (p. 14). The policy recommends the implementation of quality assurance mechanisms to ensure clinical and laboratory infrastructure and capabilities to perform genome analysis appropriately (p. 15).

In Thailand’s National Biotechnology Policy Framework (2021–2021), quality healthcare is discussed in relation to equity in access. Quality is addressed in a broad manner and is not only directed to clinical genomic services. Overall, improvement of quality of life is a main target of the policy through biotechnology. In the Thai genomic policy, there are four sectors: food and agriculture, medicine and health, bio energy and bio-based industry, and quality is prioritized in all four sectors.

#### 5.1.5 Coordination of services

The Core Concept ‘Coordination of services’ is addressed to a moderate extent in the selected policies. Mannan et al. view coordination of services as the ability of health professionals to deliver services through the established interpersonal relations, through structural mechanisms that liaise different agencies that connect different levels of local, state and federal governments in different health care systems (p. 48). Coordination of services was addressed only in 8 policies (44%) from Australia, UK, Hong Kong, Finland and Canada. The EquiFrame ratings for these policies vary from 1 to 4, the highest rating that can be given to a policy for detailing specific policy actions to achieve the Core Concept and methods to monitor the progress of the specified policy actions. While some policies acknowledge the important role that the state government or the national health services/system must play in coordinating the services (Such as the WA genomic strategy and Accelerating Genomic Policy in the NHS), others identify the ways that this can be achieved (Such as the UK Rare Disease Framework) and how integration of clinical genetic care can into routine healthcare can be achieved through coordination of services (Such as the NSW Health Genomics Strategy Implementation Plan 2021–25). None of the policies referred to any specific vulnerable groups in relation to coordination of care.

The policy that earned the highest rating for coordination of services is the WA genomic strategy (2022). The policy views the state government as the entity that would coordinate between different agencies and organizations to achieve the type of genomic care that is explained in the policy (p.10). In this policy, under Priority 1 (a person- and family-centered approach to genomic healthcare), a coordinated and personalized approach is prioritized to meet the needs, values and preferences of the consumer and the family (p. 18). In Priority 2 (genomic health services), the policy emphasizes the importance of collaborative and coordinated mechanisms in health service planning and capacity-building to integrate new genomic advancements into the WA health system (p. 23). The policy further explains this integration through the example of appropriate coordination between state-wide pathology and clinical genomic services and other healthcare providers to provide equitable and ethical genomic care to the WA population. The policy states that strong stewardship, leadership and governance are required to achieve service coordination to deliver the strategy (p. 40).

Coordination of care is a policy priority in the UK Rare Diseases Framework (2021). The policy identifies the importance of coordination of various services for rare disease management and the challenges that exist for the coordination of this care (p.14). According to the policy, care coordination helps minimize burden on patients and their carers and helps health professionals work together to provide the best care possible to the patient. The UK Rare Diseases Framework has a rating of 2 in the EquiFrame framework because the policy not only states the Core Concept but also describes and explains how it can be achieved. For example, the policy demonstrates how implementation of virtual multidisciplinary team meetings, telemedicine and video appointments have supported better care coordination in the case of rare disease management (p. 14).

Other policies from Australia, Hong Kong, UK, Finland and Canada each earned the rating of 1 as coordination of services and care is only stated or named in the policy as a strategy that sits under the overarching framework. For example, in the UK policy of Accelerating Genomic Policy in the NHS, the coordination of services and care is identified as an important task of NHS. The policy recommends the NHS achieve equity of access to genetic care by coordinating care, sharing of best practice and through driving a standardized model of delivery across the country (p. 45).

#### 5.1.6 Cultural responsiveness

The Core Concept of cultural responsiveness is addressed in six reviewed policies (35%) from Australia and the UK. Each policy except WA Genomic Strategy (2022) earned the rating of 1 as they only mentioned the importance of the concept. The WA policy further provides details of what cultural responsiveness means and how it can be achieved. Apart from the WA policy, generally cultural responsiveness is a Core Concept that is not addressed in an in depth manner in the selected policies.

While in the WA Genomic Health Strategy the term cultural responsiveness is not used, respecting ethnic, cultural and socio-economic diversity of patients and families is integral to its priority 1, achieving person and family centeredness (p.18). The policy highlights that respecting culture of Indigenous peoples is achieved by forming close partnerships with Indigenous community organizations (p. 21–22); the co-creation of genomic health services and policies therefore intrinsically recognizes the importance of community for Indigenous people and is a key aspect of cultural responsiveness. Importantly, the policy acknowledges that at present there is lack of trust within the Indigenous community towards genomic testing due to experiences of violations of their trust by early genomic initiatives. The policy therefore aims to develop standardized and culturally appropriate voluntary informed consent processes and uphold Aboriginal data sovereignty (p. 35). It also acknowledges the future need to explore the needs of consumers from various ethnic, social, and cultural groups (p. 25).

In the Australia National Health Genomics Policy Framework (2018–2021), cultural responsiveness is not addressed using the same terminology, however promoting awareness of genomics among culturally and linguistically diverse communities through culturally appropriate information is identified as a priority action. As barriers to equity are multifaceted, the policy recognizes that it is important to identify cultural acceptability barriers for genomics. Further, the policy also recognizes that evaluating the delivery of genomic health care in terms of its cultural appropriateness is an important priority action when delivering secure and responsive care to Aboriginal and Torres Strait Islander communities (p. 6).

The UK’s “Genome UK: The Future of Healthcare” (2020) policy identifies the importance of clinicians and researchers working together effectively to give individualized care for the patient which will include addressing cultural barriers (p. 26).

### 5.2 Other core concepts that are addressed

The Core Concepts of capacity building and efficiency are addressed in six policies each (35%) from Australia, Canada, UK, Denmark, Hong Kong, India and Italy. The WA Genomics Strategy (2022) received a rating of 4 for the capacity-building Core Concept and a rating of 2 for efficiency, while Australia’s National Strategic Plan for Rare Diseases (2020) received a rating of 2 for capacity building in relation to ethnic minorities. All other policies include the two concepts as principles that direct the policy; they therefore did not receive ratings for these Core Concepts.

The Core Concepts of non-discrimination, individual services, privacy and prevention are addressed in five policies each (29%). They are addressed in policies from Australia, Canada, Denmark, Thailand and Italy. In each policy the Core Concept is stated but not explained in any detail and the policy did not detail how the Core Concept would be addressed. However, all five policies acknowledge the.

The Core Concepts of liberty and entitlement are not addressed in any of the 17 policies.

### 5.3 Vulnerable communities

The coverage of vulnerable communities in the policies is sparse. Out of the 17 policies only eight (47%) specifically addressed vulnerable populations. In the other nine policies the Core Concept were addressed universally.

All five Australian genomic policies included several vulnerable populations. Reference to Aboriginal and Torres Strait Islander communities were included in The National Strategic Action Plan for Rare Diseases (2020); National Health Genomics Policy Framework 2018–2021; Genetic and Genomic Healthcare for Victoria 2021: Improving the health and wellbeing of Victorians (2021); NSW Health Genomics Strategy: Implementation plan 2021–2025 (2021); and the WA Genomics Strategy 2022–2032: Towards precision medicine and precision public health (2022). Three of the Australian policies (national plan for rare diseases, NSW genomic policy and WA genomic policy) stated specific policy actions that need to be taken to achieve better outcomes for vulnerable populations, and the WA policy also identified ways to monitor their progress.

The Australian policies also identified culturally and linguistically diverse populations and communities who are living in remote and rural areas. For example, to achieve the Core Concept of individualized services with ethnic minorities the WA genomic policy also maps out strategies to monitor the progress of the Core Concept among the minority groups. In addition, the national action plan for rare diseases also includes people suffering from chronic diseases and people with limited resources.

The other three genomic policies that include vulnerable populations are from Canada and the UK. The Canadian Strategic Plan 2022–2027: Sequencing Our Future addresses five Core Concepts in relation to its Indigenous population, ethnic minority groups and people who live in remote areas. The policy not only states the vulnerable communities but describes in detail the context of these concepts in relation to the Vulnerable Groups. Genome UK: The Future of Healthcare (2020) includes ethnic minorities in addressing three Core Concepts. The policy focuses on the importance of obtaining participation of ethnic minority groups in genomic programs and identifies specific policy actions to address barriers for these groups. The UK rare disease framework also includes the above two vulnerable populations in relation to Core Concepts such as participation, coordination of services, cultural responsiveness and access.

## 6 Recommendations

This policy review highlights both the achievements and significant deficiencies in global genomic policies. It recognizes that genomic health science is rapidly evolving, presenting a major challenge for policies to remain current and effectively address new discoveries. Many of the policies that this desktop search captured from around the world focused on clinical genetic research. There is a relative dearth of policy that focused on clinical genetic services which may reflect a gap in policy and policy research translation and implementation. Further, it may reflect that the rapidity of genomic advances is accelerating past the traditional policy implementation and review processes and that there is a need for more culturally safe and responsive approaches.

Economic, socio-cultural and financial contexts do cause the recommendations for genomic policies to vary significantly among high income to middle income and low-income countries. Particularly in middle- and low-income countries there is a notable tension between striving for universal health equity and catering to specific local needs, often relegating genomic equity to a lower priority compared to high income countries. However, it is important to acknowledge that learning of genomic policy does not have to be a one-way stream from HIs to LMICs. Strength based approaches that are used for vulnerable populations such as Indigenous communities and solutions that are developed in LMICs are robust, practical, sustainable and efficient in low resource settings and can be quite effectively translatable in HIC settings (24–26).

- National and state governments and policy makers should commit to regular genomic policy reviews to ensure they remain current.
- Policymakers need to elaborate on the principles and core concepts of equity, detailing specific actions and strategies to measure and improve access.
- Equity in access in policy needs to be addressed beyond the description of its multi-faceted nature. Policy needs to include specific actions to increase or achieve equity in access and strategies to measure it.
- Policy must actively include participation of vulnerable communities and populations at all stages from problem diagnosis to monitoring, ensuring their needs and perspectives are considered.
- Genomic policy should be integrated within broader health policies encompassing prevention, protection, and promotion.
- Special emphasis should be placed on including vulnerable populations in genomic policies. This inclusion should be comprehensive, involving co-design and stakeholder consultations to understand and address barriers effectively. Strength-based approaches need to be acknowledged when including populations such as Indigenous communities.
  - Community strengths and goals need to be starting points of health policy
  - Community perspectives of their problems, strengths and goals must inform the social construction of their need in genomic health services.
  - The goal and design of health policy including genomic policy in this case need to be centered around access, needs of the community and provision of opportunity which would empower the community meet their needs [(27). p. 119]
- Quality of care in genomic services must consider the interplay of socio-economic, cultural and geographic factors.
- Cultural responsiveness should be a key element, acknowledging the various challenges at systemic, organizational, professional and individual levels
- Genomic policy needs to explore the possibility of integrating genomics into primary health care which would increase equity in access to genomic services.
  - When delivering genomic services to vulnerable populations such as Indigenous communities, organizations such as Aboriginal Community Controlled Health Services which promote and apply culturally informed models of care, need to be involved.
  - For example, Aboriginal Community Controlled Health Services Western Australia provides support for its member services to deliver comprehensive primary healthcare using a model of care that is underpinned with values of strength in Indigenous culture, community control and self-determination.^2^
- Insights and experiences in achieving healthcare equity in HICs and LMICs can offer valuable lessons to each other.

## 7 Discussion

### 7.1 Inclusion of equity-focused core concepts in genomic policy

The results of the desktop review of genomic policy demonstrate that the concept most frequently included in relation to achieving equity in genomic healthcare in policies is ‘access’. Previous literature points to a lack of clarity in the use of the concept of access in policy literature (38). Dimensions of access focused on in the policies vary according to the country demography and priorities. According to its social, economic and cultural contexts, the national genomic policies of Australia and the UK make reference to equity in access in terms of timeliness, location, economic and financial ability, availability of genomic services, age and ethnic and cultural background. The multifaceted nature of access that the Australian and English genomic policy describe aligns with the conceptualization of access by Levesque et al. (38) which contain five dimensions: Approachability; Acceptability; Availability and Accommodation; Affordability; and Appropriateness. Their conceptualization of access further aligns with how EquiFrame describes ‘access.’ Access in EquiFrame is defined in terms of the definition of access published by the United Nations Committee on Economic, Social and Cultural Rights (2000) which refers to the need for health facilities, goods and services without any discrimination. According to their definition, access further subdivides into physical, economic and information accessibility. Mannan et al. (19) state that policy has a main role to play in addressing issues with access to health care. Braveman and Guskin (15) argue that when assessing health equity, one needs to compare how health determinants affect more advantaged and less advantaged people differently and according to them this comparison becomes a reflection of how policies are on the course to achieve or not achieve equity in a population.

In the Australian and UK genomic policies analyzed, access is addressed relative to the policies of other countries assessed in this review. They often reference the multifaceted nature of access: timeliness, location, economic and financial ability, availability of genomic services, age and ethnic and cultural background. However, in neither in the policies of Australia/UK nor of other countries, was access addressed beyond this explanation of its multiple dimensions. Specific policy actions to increase or achieve equity in access to genomic care and intensions or measurements to assess the intended improvement of access were missing in the policies.

### 7.2 Less frequently addressed core concepts

#### 7.2.1 Barriers for participation

Participation, integration, quality, coordination of services and cultural responsiveness are the principles that are addressed to varying degrees in the policies that are reviewed.

Participation is a concept that is commonly used in genomic policies particularly in high-income countries such as Australia, Canada and the UK. Participation is defined by WHO as the social involvement of the population in decisions that affect their health, defining their problem, health care services, implementation, evaluation and monitoring of those decisions (48). WHO contends that participation is a key driver of health equity as the employment of this principle facilitates the involvement of vulnerable communities as the agents and protagonists in the policies that directly affect them (2019). In the reviewed policies the terms engagement, patient voice and lived experience of patients are used interchangeably with participation. In the genomic policies where this Core Concept is addressed, there are two distinct areas that the focus is on in relation to obtaining participation of the communities: genomic research and policy implementation.

A common theme observable in the genomic policies of the UK and Australia (WA) is acknowledgement of the historical reasons for the reluctance to engage with genomic health system among vulnerable populations. This is compounded by assumptions that health professionals have about minority communities. For example, previous literature demonstrates that health professionals make assumptions about socio-cultural, economic and biological characteristics of ethnic and racially different minorities that effects their care (28). This two-way barrier has led to a lack of diversity in available datasets across high income countries, which in turn impacts negatively on the ability of minority populations to obtain benefits of genomic medicine and healthcare (2).

To increase participation in genomic research or care from ethnic and other minority groups, genomic policies need to recognize and address this two-pronged barrier effectively. The ‘Accelerating Genomic Medicine in the NHS’ (43) policy sets forth a model to follow to achieve this outcome. In the reviewed genomic policies, there is an absence of recognition and weight given to the capacity that participation has, to function as a driver for equity.

Participation is mentioned in the reviewed policies mainly in relation to research and policy implementation. However, participation as a strategy to achieve equity in genomic health care needs to be included from the first step itself of policy development including in research and policy implementation. This brings our attention to an important measure that needs to be taken in future policy making in genomic health care: the need to include participation of vulnerable communities and populations in all stages of policy process that includes the stages of problem diagnosis, planning, implementation, evaluation and monitoring. Health inequity and inefficiency are significant negative consequences that irrefutably follow, in the instance when this is not done (48).

#### 7.2.2 Integration as a strategy for equity

Integration of genomic healthcare in the mainstream health system is the most notable strategy and service model that many policies recommend in relation to the Core Concept of integration. Integration of health services is recognized as a mechanism that facilitates and enhances health equity (49). WHO contends that integration as a principle encourages the prioritization and selection of health services to deliver a holistic care over the life course of a population that include processes related to prevention, protection, promotion, diagnosis, treatment and management of diseases (2018).

Regarding the principle of integration, Australian and English genomic policy in particular read strongly according to the EquiFrame framework: they all focus on giving clearly articulated, specific policy actions to integrate genomic healthcare into the mainstream health system. Within the principle of integration, Australian policies identify focus areas that need to be included in it such as the inclusion of education, workforce knowledge about genomics, data security and sharing, high quality and safe services and person-centered approach as strategies to employ. These aspects of integration are also endorsed by WHO (49). The examination of integration in Australian genomic policy provides a relatively complete model of the ways and steps that can be taken to achieve genomic care equity through integration. While the integration of the above sectors is important in genomic health care, it is also important to acknowledge the need for integration of the overarching processes of prevention, protection, and promotion. The developments of genomic medicine and healthcare have a significant impact on these processes.

#### 7.2.3 Quality of care and equity

Quality of care is integral to health equity. In fact, effectiveness, people centeredness, equity, timeliness, safety, integration and efficiency are widely recognized to be the characteristics of quality of care (29). There is a growing body of literature that argue that quality of care is achieved only if it is examined through an equity lens (30). Equity and quality of care in health services should not depend on age, sex, gender, race, indigeneity and ethnicity, migrant status, geographical location, socio economic status, language, religion and disability and other socio-economic and cultural factors (30, 31). The delivery of equitable and high-quality health care demands an understanding of the complex interplay of the above factors (30). The reviewed policies recognize quality of care as an outcome of strong senior leadership in the health system and an outcome of person-centered genomic healthcare, improved safety, cost effectiveness and of a skilled and knowledgeable health workforce. However, in these policies, there is little acknowledgment given to the understanding of the interplay of various socio-economic, cultural and geographic aspects in relation to achieving quality of care in genomic healthcare.

#### 7.2.4 Cultural responsiveness as an afterthought

Cultural responsiveness is one of the Core Concepts that is sparsely addressed in the reviewed policies. Naturally, cultural responsiveness becomes an important necessity in health policy where there is a significant culturally diverse population; this Core Concept is seen in most Australian and English genomic policies. Forming close relationships with the communities of Indigenous and ethnic minorities, developing culturally appropriate voluntary informed consent processes, increasing cultural awareness in genomic clinicians and researchers are some of the policy actions that can be seen in them to increase cultural responsiveness. Recognition of culture is a cornerstone of strength-based approaches used to achieve health equity in marginalized and vulnerable communities (27). Cultural responsiveness is an important Core Concept that needs to be included in genomic policies as literature has already demonstrated that the assumptions that health professionals tend to make of patients from minority communities function as a significant barrier for accessing genomic healthcare (28). One’s culture and language act as major factors that impact on the quality of the healthcare a patient receives, health outcomes and patient satisfaction (36). Although cultural diversity and cultural competence are recognized as important obligations that the health services need to meet to address the needs of ethnic minorities and Indigenous communities, literature identify systemic, organizational, professional and individual challenges that health services encounter when delivering culturally responsive care (37). Genomic policies that were reviewed give little focus on the importance to the cultural responsiveness when addressing the needs of ethnic minority and Indigenous communities.

Other Core Concepts such as prevention, liberty and entitlement were either addressed in very few policies or were not addressed at all. However, all three concepts are intrinsically linked to the provision of genomic care and to factors such as confidentiality, privacy and data sovereignty.

### 7.3 Missing CCs and implications

Liberty and entitlement are not included in any of the selected policies. However, the gravity and the negative consequences of historical exclusion of the CC of liberty, especially from genomic policies related to vulnerable groups such as Indigenous populations and ethnic minorities, are clearly documented in the selected policies. Liberty for example is defined by Turnbull and Stowe (32) as a constitutional principle that gives the right for persons for certain entitlements and freedoms that include physical and general freedom to conduct one’s life as one prefers to. Considering the historical damaging of trust that has taken place due to unwarranted and unconsented genetic initiatives with Indigenous people in Australia, it would be detrimental and harmful for not including the CC of liberty in future genomic policy.

According to Turnbull and Stowe (32) entitlement is one’s right to receive services according to one’s strengths and resources, to be served for one’s benefit to the utmost extent possible. Entitlement is a CC that could be considered as indispensable for genomic policies, especially at present time where irrespective of vulnerability, awareness of genomic services is considered low in the general populations. According to Goddard and Smith (33) the CC of entitlement would not be present in a situation where there is a lack of awareness and clarity of the availability of a specific health service by all population groups. Therefore, entitlement is a CC that future policy reviews of genomics will have to focus on especially in contexts where there is unequal awareness and knowledge of genomics services in the population.

### 7.4 Vulnerable communities

Only about a quarter of the reviewed policies included vulnerable communities; most policies mentioned the Core Concepts universally. The most noted vulnerable communities in the policies are Indigenous and CALD communities and communities that live in rural and remote areas. Although in many contexts these populations also overlap with economically and financially deprived groups, the exclusion of the latter in policy could result in such vulnerable groups being overlooked by services.

Recent literature on vulnerability and vulnerable groups emphasizes the importance of promoting an intersectionality framework to use as a guiding principle to study vulnerable individuals and groups because vulnerability is a nuanced concept and cannot be rigidly applied to all in a socio-demographic group (50). However, in EquiFrame framework this nuanced nature of vulnerability of groups of people is not captured.

This policy review focuses on the level of inclusion of equity in genomic policies mainly in high income countries such as Australia, England, Canada, Hong Kong, Finland and Denmark. Genomic policies are not common in middle and low income and developing countries. This demonstrates that genomic health is still an area in healthcare that is limited to the high-income countries and there is a long and windy road ahead of low- and middle-income countries to include the benefits of discoveries made in genomic care in health policy.

However, it is important to acknowledge that there is a significant body of literature that point out to the effectiveness and translatability of many low cost and high impact innovations that are employed in LMICs especially to tackle their high health care costs which could be adopted to address problems such as inequity in health in HICs (24–26, 34).

### 7.5 Limitations

Although EquiFrame is a practical tool that policymakers and reviewers can use to assess a policy, it also presents few limitations. Even though the framework was developed to review policy in low- and middle-income countries, the policies reviewed in this article come mostly from developed, high income countries. Therefore, in relation to the Core Concepts, there could be others we may have missed that are only captured in policy from developed countries. The other limitation is the inclusion of vulnerable populations. The identification and determination of vulnerable population groups could vary widely in different contexts (35). For example, Ivanova et al. (22) argue that identification of a vulnerable group is a complex process as there is no single criteria or indicator to benchmark or measure vulnerability of a group of population (22). Genomics is a highly specialized field of health service. Colonial histories and related issues such as racism and discrimination render certain groups such as Indigenous populations particularly vulnerable and could make them mistrust services that offer genetic services (51). Therefore, to capture these populations, we have included Indigenous population group in addition to the given vulnerable groups to make the list more inclusive.

Further, even though law, legislation and acts can have a direct impact on achieving equity in health services, we have excluded them from this review, due to their varied and multitude nature across countries and the lack of easy accessibility. We sought to contain this analysis only to policies of genomic services. Therefore the exclusion of laws, legislations and acts from this review can also be considered as a limitation.

## 8 Conclusion

This policy review reveals that with few exceptions, genomic policies across high or middle-income countries fall significantly short in outlining specific, actionable steps toward achieving equity. Given that genomics is an evolving field with rapid advancements in discoveries and clinical applications, it is understandably challenging for policies to stay abreast of these developments. Ensuring that the benefits of these advancements are equitably distributed poses a further challenge. Therefore, policymakers are tasked with the dual responsibility of not only keeping policies current with the fast-paced evolution of genomics but also ensuring these advances are accessible and beneficial to all, thereby maintaining a balance between innovation and equity in genomic healthcare.

## Data Availability

The raw data supporting the conclusions of this article will be made available by the authors, without undue reservation.

## Footnotes

^1^ http://prisma-statement.org/

^2^ https://www.ahcwa.org.au/about-us/
