# Supplementary figures and images for: “Equity” in genomic health policies: a review of policies in the international arena
Source: Front Public Health. 2024 Dec 20;12:1464701. doi: 10.3389/fpubh.2024.1464701 (PMC11695411; doi:10.3389/fpubh.2024.1464701)

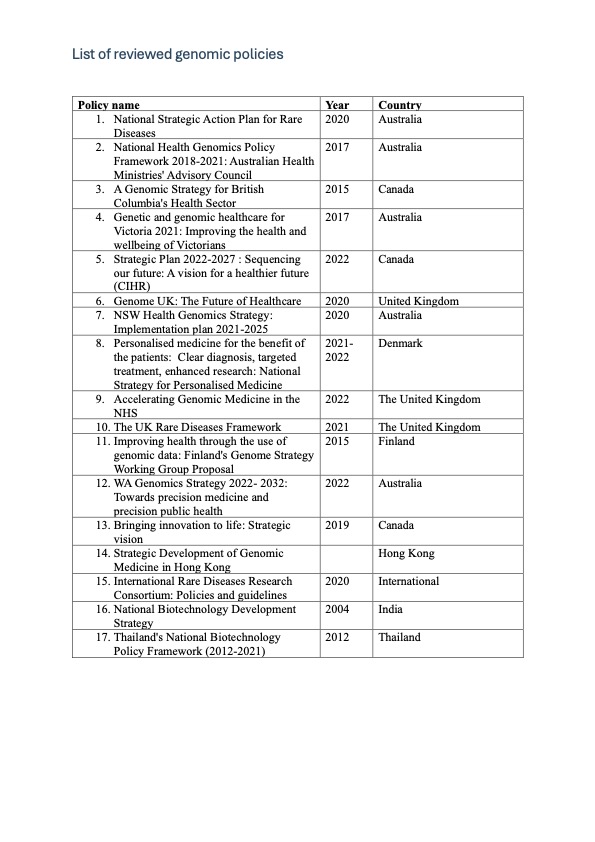

Supplement: Supplementary file 1 [file Image_1.jpeg]

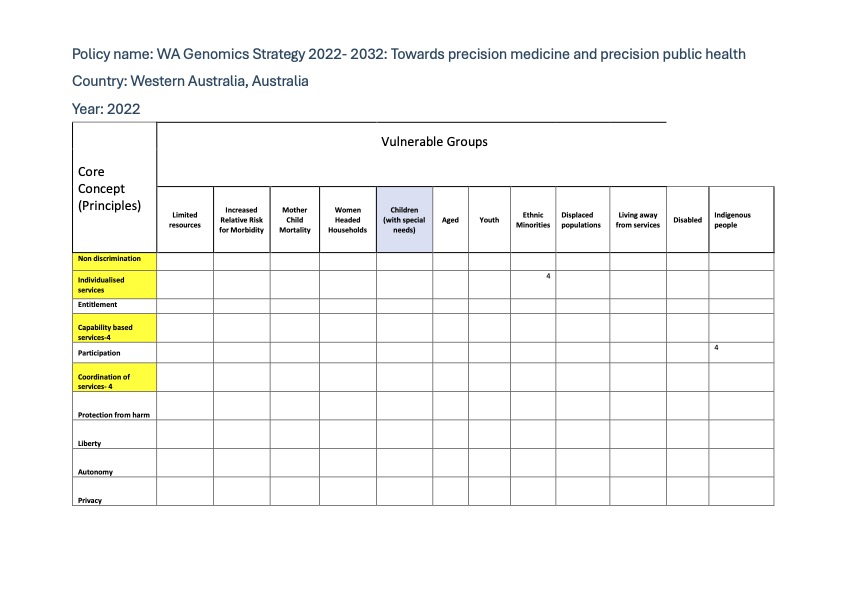

Supplement: Supplementary file 2 [file Image_2.jpeg]
